# Characterization of higher harmonic modes in Fabry–Pérot microcavity organic light emitting diodes

**DOI:** 10.1038/s41598-021-87697-8

**Published:** 2021-04-19

**Authors:** Ekraj Dahal, David Allemeier, Benjamin Isenhart, Karen Cianciulli, Matthew S. White

**Affiliations:** 1 Materials Science Program, University of Vermont, Burlington, VT 05405 USA; 2 Department of Physics, University of Vermont, Burlington, VT 05405 USA; 3 Asheville School, Asheville, NC 28806 USA

**Keywords:** Materials for devices, Electronic devices, Organic LEDs, Lasers, LEDs and light sources

## Abstract

Encasing an OLED between two planar metallic electrodes creates a Fabry–Pérot microcavity, resulting in significant narrowing of the emission bandwidth. The emission from such microcavity OLEDs depends on the overlap of the resonant cavity modes and the comparatively broadband electroluminescence spectrum of the organic molecular emitter. Varying the thickness of the microcavity changes the mode structure, resulting in a controlled change in the peak emission wavelength. Employing a silicon wafer substrate with high thermal conductivity to dissipate excess heat in thicker cavities allows cavity thicknesses from 100 to 350 nm to be driven at high current densities. Three resonant modes, the fundamental and first two higher harmonics, are characterized, resulting in tunable emission peaks throughout the visible range with increasingly narrow bandwidth in the higher modes. Angle resolved electroluminescence spectroscopy reveals the outcoupling of the TE and TM waveguide modes which blue-shift with respect to the normal emission at higher angles. Simultaneous stimulation of two resonant modes can produce dual peaks in the violet and red, resulting in purple emission. These microcavity-based OLEDs employ a single green molecular emitter and can be tuned to span the entire color gamut, including both the monochromatic visible range and the purple line.

## Introduction

Organic light-emitting diodes (OLEDs) constitute a promising, next-generation display technology with significant market share, largely due to the ability to tune emission color by changing the molecular structure^1,2^. The microcavity OLED employs two reflective surfaces, either two metallic electrodes or one dielectric mirror, which limits the emission to narrow-band resonant cavity modes due to exciton-photon coupling^3–8^. Compared to the broadband emission of conventional OLEDs^2^, the emission of microcavity OLEDs offer potential advantages for spectroscopy, optical communications and displays^6,9–12^. The thickness of the transport layers can be varied to tune the emission through the visible^13^ and into the deep blue and ultraviolet^14^, a color range that has proven challenging for conventional OLEDs.

A microcavity OLED with two conducting plane mirrors is a Fabry–Pérot etalon in which specific wavelengths of light resonate while others destructively interfere and are eliminated. The mirrors act as cathode and anode, between which the electron transport layer (ETL), the emissive layer (EML), and the hole transport layer (HTL) of the OLED occupy the volume of the etalon. This simple, elegant device design produces remarkable optical properties but is bound by the coupling of the optical pathlength to the resistive loss in electrical conductivity through the low-mobility organic semiconductors. The cavity has an infinite series of discrete integer half-wavelength resonant modes due to the confining nodes at the boundaries that satisfy:

1 $$\begin{aligned} \frac{j\lambda _j}{2}=nd\quad j=1, 2, 3, \dots \end{aligned}$$

where *n* is the index of refraction, *d* is the thickness of the active OLED layer, and $\lambda _j$ is the resonant wavelength of the $j^{\mathrm{th}}$ mode. The resonant behavior is much like stringed instruments, closed tubes, or particle-in-a-box square wells, in which the presence of hard boundaries enforces zero wave amplitude at particular locations. The goal of this work is to address a range of these modes in the microcavity OLED, which requires that the molecular emitter has some electroluminescence intensity at the resonant wavelength. Thin-film OLEDs are ideally suited to address the fundamental resonant mode ($j=1$) with emission peaks in the visible range between 400 and 700 nm, since the thickness (*d*) of an OLED is roughly 100 nm, and the index of refraction (*n*) of the organic semiconductors is generally between 1.5 and 2^3,4,13,14^. The optical path length within such an OLED microcavity is therefore around 200 nm, giving a fundamental, $\lambda /2$ resonant peak at 400 nm. In order for the OLED to pump the higher order modes ($j=2, 3, \dots$), either the OLED must have significant UV electroluminescence intensity or the optical pathlength of the cavity must be doubled, tripled, etc. Extending the optical path length reduces the device electronic efficiency and increases the power consumption and waste heat dramatically due to increased resistance^15,16^. Without a heat sink, this resistive Joule heating can lead to steady-state temperatures of roughly $85^\circ \hbox {C}$ at a power consumption of $0.1\,\hbox { W/cm}^2$^17^. In this work, we employ a Si-wafer substrate to act as a built-in heat sink to enable high-power operation of microcavity OLEDs with 100–350 nm thickness. This range allows for the EML semiconductor Tris(8-hydroxyquinolinato)aluminum ($\hbox {Alq}_3$) to directly pump the fundamental and first two higher harmonics, revealing a larger picture of the cavity mode structure.

## Materials and methods

### Materials

The organic semiconductor combination consisting of tris-(8-hydroxyquinoline)aluminum ($\hbox {Alq}_3$) as EML, bathophenanthroline (BPhen) as an ETL, and *N*,*N*′-di(1-naphthyl)-*N*,*N*′-diphenyl-(1,1′-biphenyl)-4,4′-diamine (NPB) as a HTL, was selected as a well-known standard 3-layer green OLED device stack^18,19^. The organic semiconductors were purified by vacuum sublimation. Silver (Ag) and molybdenum oxide ($\hbox {MoO}_x$) were used for the anode, while aluminum (Al) and lithium flouride (LiF) were used for the cathode. The $\hbox {Alq}_3$, BPhen, NPB, $\hbox {MoO}_x$, LiF were purchased from Sigma-Aldrich and Ag, Al were purchased from R.D. Mathis.

**Figure 1:**
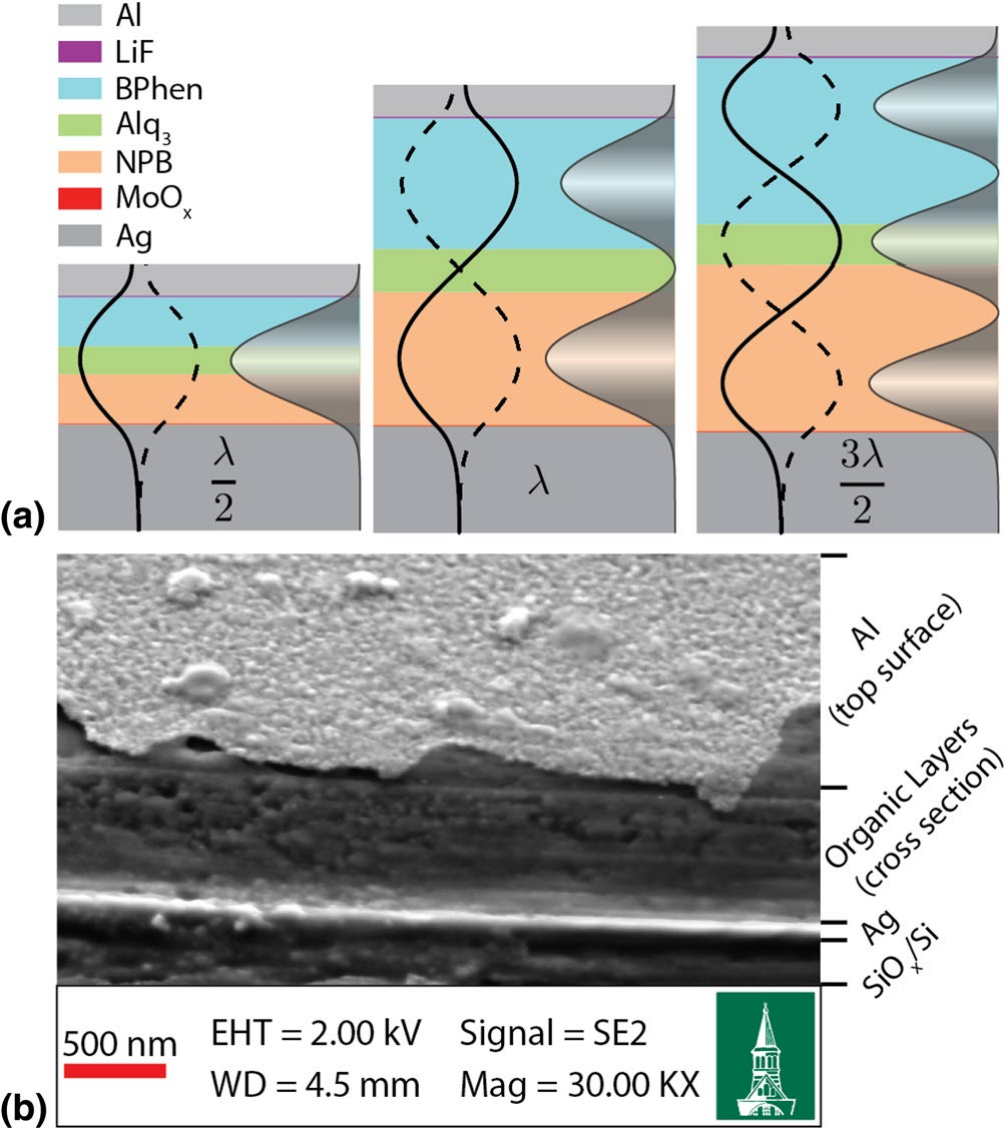
(**a**) Schematics of $\lambda /2$, $\lambda$, and $3\lambda /2$ resonant modes in microcavity OLEDs. Materials with representative thickness in the schematics are color-coded. The standing-wave electric field and the intensity are computed (as described below) for the observed resonant wavelength and plotted to illustrate the three resonant modes. (**b**) Cross-sectional SEM image of a 308 nm thick microcavity OLED. Cross section was prepared by scribing the surface and is not a vertical cross section. Image taken at $45^{\circ }$ tilt shows top cutaway organic layers and top surface of the 30 nm thick Al top electrode.

### Device fabrication

A standard OLED recipe with the substitution of a 30 nm Al semitransparent metal electrode in the place of a transparent electrode (ITO) was used in order to generate optically resonant microcavities^18,19^. A sequential thermal evaporation of 100 nm bottom electrode of Ag, followed by 1 nm of $\hbox {MoO}_x$, *a* nm of NPB, *b* nm of $\hbox {Alq}_3$, *a* nm of BPhen, 1 nm of LiF, and 30 nm of Al was performed on an oxidized silicon substrate. Table 1 shows the thicknesses of the ETL/HTL and EML for each microcavity thickness. The total organic layer thickness was measured on a Dektak XT stylus profilometer sampling 5–10 locations on the $3\,\hbox { cm}^{2}$ organic film ($1.74\,\hbox { cm }\times 1.74\,\hbox { cm}$). Device active area was defined by intersecting 1 mm wide metal electrodes, resulting in a $1\,\hbox { mm}^2$ device area. All depositions were performed under a pressure of $10^{-7}$ Torr without breaking the vacuum during processing. The evaporation rates for the organics were 0.5 Å/s, 0.3 Å/s for $\hbox {MoO}_x$, 0.1 Å/s for LiF, 1.5 Å/s for Al, and 1.0 Å/s for Ag. The distance from the source to film was roughly 35 cm and the substrate holder was rotating at 10 rpm to ensure uniformity of film thickness. The deposition rate and thickness were measured by quartz crystal monitor in the Angstrom Engineering thermal evaporator. These devices were stored and tested in a nitrogen glove box, with $<0.1$ ppm $\hbox {O}_2$ and $<0.5$ ppm $\hbox {H}_2\hbox {O}$.

**Table 1:** OLED layer thicknesses (nm).

Total	NPB	Alq$_3$	BPhen
ITO OLED			
$100\pm 1.5$	40	20	40
Microcavity OLED			
$99\pm 0.5$	34.5	20	34.5
$101\pm 0.4$	40.5	20	40.5
$106\pm 0.9$	43	20	43
$113\pm 0.7$	46.5	20	46.5
$144\pm 1.5$	62	20	62
$182\pm 1.2$	71	40	71
$230\pm 0.9$	95	40	95
$252\pm 3.8$	106	40	106
$267\pm 2.6$	113.5	40	113.5
$282\pm 2.3$	121	40	121
$304\pm 2.0$	132	40	132
$324\pm 4.2$	142	40	142
$334\pm 1.2$	147	40	147
$354\pm 1.1$	157	40	157
$362\pm 3.2$	161	40	161

### Device characterization

**Figure 2:**
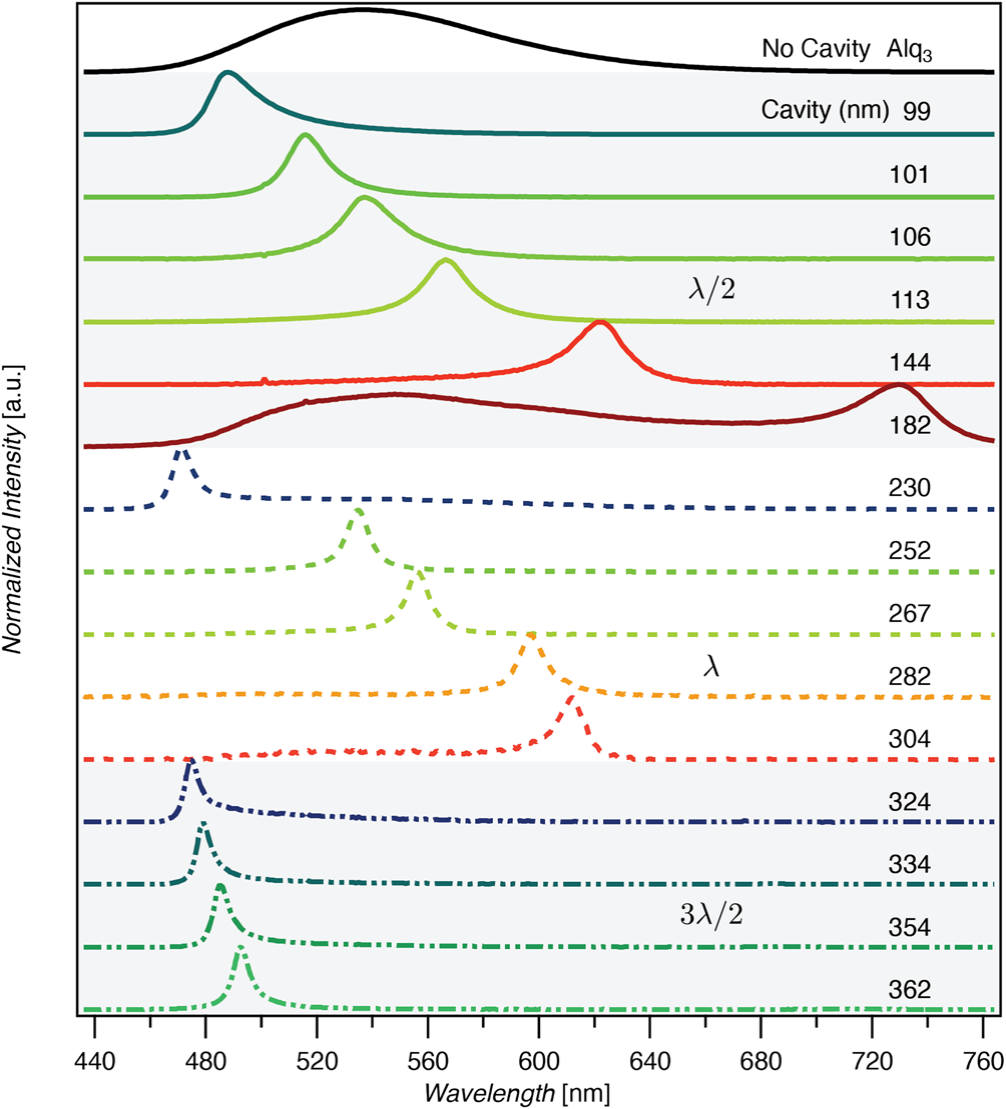
Electroluminescence spectra in the forward (normal) direction for an $\hbox {Alq}_3$ ITO-based OLED and for microcavity OLEDs with cavity thicknesses spanning 99–362 nm. The peak intensity for each spectrum is normalized.

The electroluminescence spectrum was measured using an Ocean Optics HDX or Red Tide visible and near-IR spectrometer with a fiber optic feed-through to the glovebox. The emission of the devices was passed through an iris to control the collection angle, and then reflected off of a $90^\circ$ off-axis parabolic mirror to focus the emission into the fiber. The iris has adjustable diameter from 0.8 to 12 mm at 17 cm distance from the sample. 12 mm diameter was used collecting spectra for Figs. 2 and 4. The ARES pattern was measured at every $1^{\circ }$ intervals from $0\hbox { to }70^{\circ }$ angle by mounting the device on a rotating stage. The current-voltage characteristics were measured using Keithley 2461 source meter.

## Theory

### Microcavity OLED design

The microcavity OLEDs consist of a three-layer organic semiconductor stack: NPB as HTL, $\hbox {Alq}_3$ as EML, and BPhen as ETL with a $\hbox {Ag/MoO}_x$ anode and an Al/LiF cathode. The Al cathode is 30 nm thick and therefore functions as a semitransparent mirror for the top-emitting microcavity OLED. A silicon wafer substrate provides both planarity for high reflectivity mirrors and a thermal conductivity roughly 100 times that of glass, allowing the substrate to effectively dissipate excess heat away from the microcavity during high-power operation^20,21^. A 100 nm $\hbox {SiO}_x$ layer is grown on the wafer surface to provide electrical insulation^22^. Schematic illustrations of the device structure are shown in Fig. 1a, representing the first three resonant modes of the microcavity. The electric field standing wave (solid and dashed lines) and the field intensity (grayscale gradient) are calculated for the observed resonant wavelength(s) of progressively thicker cavities using the transfer matrix technique described below. The quantitative field profiles can be seen in Supplementary Figure S1. This illustrates the experimental design employed in this work: increasing the thickness of the ETL and HTL layers widens the spacing between the two mirror electrodes, allowing the visible electroluminescence from $\hbox {Alq}_3$ to pump these three modes. Figure 1b shows an SEM image of a 308 nm thick microcavity OLED (scribed cross-section, $45^{\circ }$ sample tilt), where the organic active layers can be seen between the metallic electrodes, with the top surface of the 30 nm Al cathode sloping away in the top of the image.

The microcavity effect suppresses the broadband electroluminescence of $\hbox {Alq}_3$, producing narrow-band, angle- and polarization-dependent emission peaks where the emission intensity depends on both the transmissivity of the structure and the free-space emission intensity at the resonant wavelength. We begin with the classical treatment of an ideal Fabry–Pérot etalon to explore the resonant modes of the microcavity OLED. The selectivity of light through a lossless Fabry–Pérot etalon at normal incidence is given by:

2 $$\begin{aligned} T(\phi )=\frac{I_{\mathrm{trans}}}{I_{\mathrm{inc}}}=\frac{(1-R_1)(1-R_2)}{(1-\sqrt{R_1R_2})^2+4\sqrt{R_1R_2}\sin ^2\phi } \end{aligned}$$

where $\phi$ is the phase shift between forward and backward propagating waves inside the etalon, and $R_1$ and $R_2$ are the reflectivity of the top and bottom mirrors. The phase shift $\phi$ between the two mirrors is related to the optical path length of the device and the wavelength of light $\lambda$:

3 $$\begin{aligned} \phi =\frac{2\pi nd}{\lambda } \end{aligned}$$

The resonant mode condition requires the phase change during one round trip to be a multiple of $\pi$ ($\phi =j\pi ,\ j=1, 2, 3, \dots$) so that $\sin \phi =0$ and the transmissivity is maximum:

4 $$\begin{aligned} T(\phi )=\frac{(1-R_1)(1-R_2)}{(1-\sqrt{R_1R_2})^2} \end{aligned}$$

This condition provides a relationship between the device thickness and the resonant wavelength, and we will refer to the first three resonant modes $j=1,\ 2,\ 3$ by the corresponding fractional wavelengths: $\lambda /2,\ \lambda ,\ 3\lambda /2$.

The emission spectrum in the forward direction (perpendicular to the planar mirror surface) can be calculated from classical optics by^23,24^:

5 $$\begin{aligned} |E(\lambda )|^2=\frac{(1-R_1)[1+R_2+2\sqrt{R_2}\cos (\frac{4\pi x}{\lambda })]}{1+R_1R_2-2\sqrt{R_1R_2}\cos (\frac{4\pi L}{\lambda })}|E_f(\lambda )|^2 \end{aligned}$$

where $E_f(\lambda )$ is the free-space molecular emission spectrum, *x* is the effective distance of the emitting layer from the top mirror, and *L* is the total optical length of the microcavity device. Considering a substantial penetration depth into the mirrors, *L* can be expressed as^24^:

6 $$\begin{aligned} L=\Big |\frac{\phi _{\mathrm{Ag}}+\phi _{\mathrm{Al}}}{4\pi }\lambda \Big |+\sum _i n_it_i \end{aligned}$$

where $n_i$ and $t_i$ are the refractive index and the thickness of organic layers, and $\phi _{\mathrm{Ag}}$ and $\phi _{\mathrm{Al}}$ are the phase changes at the silver and aluminum mirrors.

### Computational modeling of microcavity OLED emission

The solution for an ideal etalon is useful for understanding the resonant modes; however in multi-layer organic thin films this description of the etalon reflectivity is generally not sufficient to accurately predict the light emission and does not extend easily to off-normal emission. Partial reflections from differences in index of refraction at the material interfaces can contribute substantially to the observed optical behavior, particularly at large angles. In order to fully describe these multi-layer structures, a full treatment using the transfer matrix method is necessary.

The transfer matrix method is a commonly used formulation of the interaction of electromagnetic waves with 2-dimensional planar structures. The technique utilizes the Fresnel coefficients and the optical properties of the layers to relate the incident, reflected, and transmitted waves on either side of a multi-layer stack^25^. A numerical simulation program was developed to simulate the light emission from our OLED microcavities which implements the transfer matrix method modified with source terms as outlined by Benisty et al.^26^. For a concise summary of the transfer matrix technique, interested readers are directed to Petterson et al. or Berreman^25,27^.

## Results

### Electroluminescence of microcavity OLEDs

#### Emission normal to the microcavity

The forward emission spectra of microcavity OLEDs with fifteen different cavity thicknesses ranging from 99 to 362 nm are shown in Fig. 2. The emission spectrum of an ITO-based OLED with $\hbox {Alq}_3$ as emitter is shown (top spectrum, solid black line) for reference. The 99 nm thick cavity shows electroluminescence with a peak emission at 488 nm and a full width at half maximum (FWHM) of 23 nm, compared to a FWHM of roughly 100 nm for the non-microcavity OLED. As the cavity becomes progressively thicker to 182 nm, the emission peak red-shifts through the visible spectrum with comparable FWHM. These emission peaks represent the overlap of the $\lambda /2$ cavity mode with the $\hbox {Alq}_3$ luminescence spectrum. At a cavity thickness of 182 nm the resonant mode peak lies at 730 nm, a wavelength where $\hbox {Alq}_3$ has virtually no luminescence intensity. Nonetheless, the microcavity forces electroluminescence into this wavelength range through resonant coupling to the $\lambda /2$ cavity mode.

The 182 nm cavity spectrum also exhibits a broadband emission peak centered at 548 nm, reflecting the free-space emission spectrum of $\hbox {Alq}_3$. This emission warrants explanation. The broadband emission was likely not present in the microcavity, but was instead due to edge effects. On a few (less than 20%) of the microcavity OLEDs, insufficient contact pressure between the shadow mask and substrate during Al evaporation led to small, tapering edges of the electrode. While small in area, these thinner mirror surfaces were more transparent and emitted with broadband character. This edge leakage is only apparent in the 182 nm microcavity as the resonant emission is comparably weak. The device appears to the eye as red, with thin orange lines along the pixel edges.

Increasing the cavity thickness even further allows direct pumping of the $\lambda$ and $3\lambda /2$ modes. Microcavities with thickness ranging from 230 to 304 nm show $\lambda$ mode resonant peaks spanning the visible spectrum with FWHM between 9 and 13 nm. The thickest microcavities allow the blue tail of the $\hbox {Alq}_3$ emission spectrum to pump the $3\lambda /2$ mode and have 324 to 362 nm between the two reflective mirrors. Each of these emission peaks has FWHM of roughly 7 nm.

**Figure 3:**
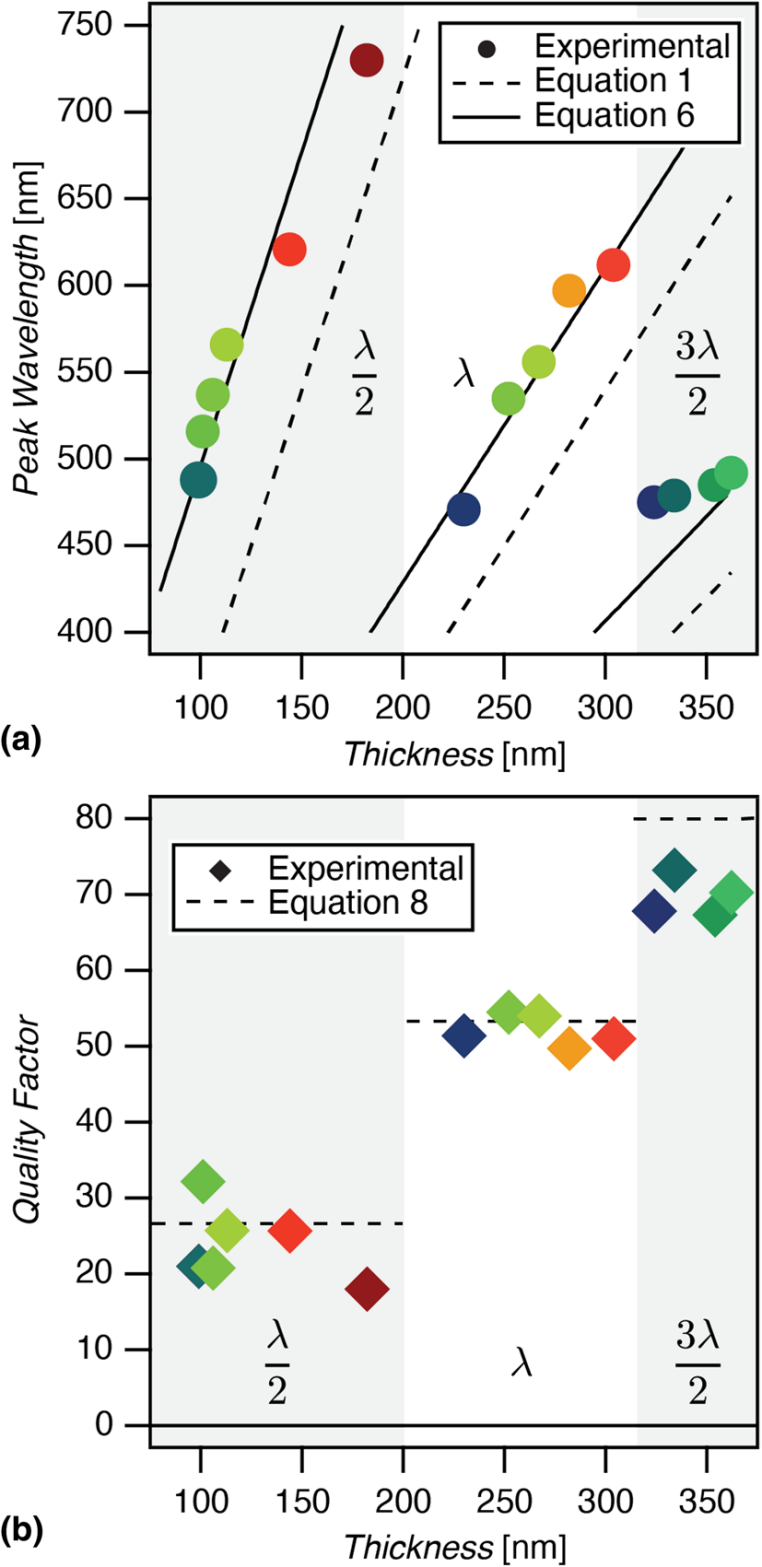
(**a**) Peak emission wavelength vs. thickness. Dashed line represents the ideal classical theory Eq. (1) with assumed index of refraction $n=1.8$, and solid lines include penetration depth of 40 nm and 27 nm in the Ag and Al using Eq. (6). (**b**) Quality factor versus microcavity thickness. Dashed lines are calculated using Eq. (8) assuming $R_1\times R_2\approx 0.8$ (see Supplementary Figure S4).

The peak wavelength of the normal emission spectra and the FWHM of the emission peaks show a strong relationship with the cavity thickness. As predicted by Eq. (1), the peak emission wavelength of the cavity mode shows a linear dependence on the cavity thickness, and the slope of the linear trend is inversely proportional to the mode index $j=1,\ 2,$ and 3. Higher-order modes are therefore less sensitive to variations in thickness. This trend can be seen in Fig. 3a, where the peak emission wavelength normal to the microcavity structure is plotted as a function of cavity thickness.

While the general trend observed in Fig. 3a matches the ideal classical theory, our microcavities do not represent ideal Fabry–Pérot etalons and demonstrate some deviations from the expected behavior. As shown by the calculated internal electric fields in Fig. 1a, the electric field in each cavity decays over a finite region of the Ag and Al reflective mirrors. The field decay length is a non-negligible fraction of the resonant wavelength due to the extremely small distance between the mirrors in the microcavities. This results in deviation from the ideal linearity and slope defined in Eq. (1), particularly near the mode minima. Our results indicate asymptotic behavior at the lower boundaries of the modes due to the coupled dependence of the skin depth and the resonant wavelength^28,29^. This interdependence is due to the continuity of the electric field and its first derivative at the interfaces in accordance with Maxwell’s equations.

The higher order modes also display significant bandwidth narrowing with increasing *j*. The trend is best described in context of the quality factor, defined as:

7 $$\begin{aligned} Q=\frac{\lambda _0}{\lambda _{1/2}} \end{aligned}$$

where $\lambda _0$ is the resonant wavelength and $\lambda _{1/2}$ is the FWHM of the emission peak. The steady-state solution of the ideal Fabry–Pérot cavity gives:

8 $$\begin{aligned} Q=j\Bigg [\frac{\pi (R_1R_2)^{1/4}}{1-\sqrt{R_1R_2}}\Bigg ] \end{aligned}$$

which indicates that the quality factor depends only on the cavity mode index *j* and the reflectivity of the metal electrodes. Figure 3b shows the quality factor of each of the microcavity OLED spectra shown in Fig. 1 as a function of cavity thickness. The quality factor shows stepwise dependence on the mode index, and the aggregate data show the ratio $Q/j=24.4\pm 3.3$.

#### Angle resolved electroluminescence spectroscopy

**Figure 4:**
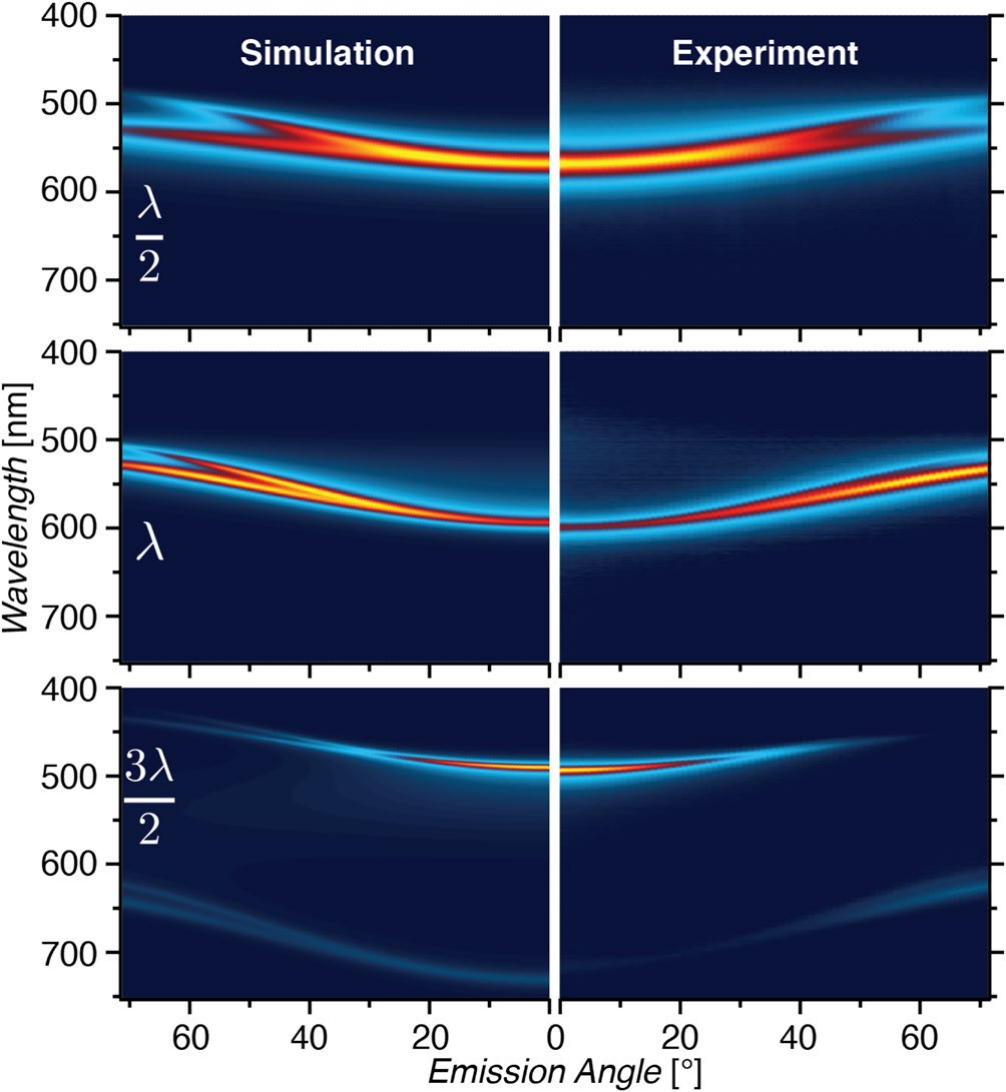
Experimental (right) and simulated (left) ARES data for cavity thickness 113 nm representing the $\lambda /2$ mode, 282 nm the $\lambda$ mode, and 362 nm the $3\lambda /2$ mode.

Light emission from the top surface of the microcavity OLED shows strong angular dependence, manifest as a visible color shift at different observation angles. Angle-resolved electroluminescence spectroscopy (ARES) reveals the emission of the microcavity OLEDs at various angles with respect to the normal emission described above. Figure 4 shows the experimental and simulated ARES data for three microcavities representative of the $\lambda /2$, $\lambda$, and $3\lambda /2$ resonant modes. Data along the central vertical axis, the $0^{\circ }$ emission angle of the experimental plots, represents the same spectra shown in Fig. 2 for devices with 113 nm, 282 nm, and 362 nm cavity thicknesses.

The ARES data reflects the outcoupling of the waveguided modes within the microcavity as a function of the emission angle. Within the microcavity, the propagating modes have resonant wavelength that depends on the angle:

9 $$\begin{aligned} \frac{j\lambda _j}{2}=nd\cos \theta \qquad j=1, 2, 3, \dots \end{aligned}$$

which converges to Eq. (1) for the resonant mode normal to the cavity axis at $\theta =0$. Figure 4 shows the resonant emission shifting toward shorter wavelengths with increasing viewing angle. The outcoupling of light from the microcavity is determined by the polarization-dependent Fresnel equations, with two significant effects on the emission spectra at high angles: a deviation from the proportionality $\lambda _j\propto \cos \theta$ and a swallow-tail splitting of the emission mode into two peaks with orthogonal polarization^11,30^. The swallow-tail splitting results from the different boundary conditions at the conducting top mirror enforced by Maxwell’s equations for the s and p-polarizations. For Fabry–Pérot microcavities, this effect is primarily due to the polarization-dependent phase shift at the metal surfaces^11^. The higher energy emission at a given angle is s-polarized and the lower energy emission is p-polarized, representing the TE and TM modes respectively. This is confirmed by inserting a linear polarizer in the detector path at $0^{\circ }$ or $90^{\circ }$ with respect to the viewing angle axis of rotation, as seen in Supplementary Figure S3.

The computational model described in “Computational modeling of microcavity OLED emission” was used to model the ARES patterns of the microcavity devices and the results are shown on the left side of Fig. 4. The model predicts all of the experimental observations, including the dependence of the emission peak-wavelength, bandwidth, and polarization on the cavity thickness and the viewing angle. This model represents a robust simulation tool for predicting the emission from emitting dipoles interacting with multiple dielectric and metallic planes, and will be made publicly available upon request.

The emission intensity is generally strongest in normal emission, trending lower at higher viewing angle. However, the $\lambda$ mode ARES pattern in Fig. 4 shows a peak of comparable intensity at $55^{\circ }$ emission angle with peak-wavelength of 537 nm, compared to the 586 nm peak at normal $0^{\circ }$ emission. There are two potential explanations for this phenomenon. The outcoupling of light at various angles is strongly dependent on the orientation of the transition dipole moment in the $\hbox {Alq}_3$ emitter layer, which can lead to weak normal emission and strong emission peaks at angles around $60^{\circ }$ when dominated by vertically oriented dipoles. However, in this case we see no inherent reason that the dipole orientation should be significantly different from the thinner and thicker $\lambda /2$ and $3\lambda /2$ microcavities shown, which do not show a secondary maximum. In this case, we believe that this intensity peak at $55^{\circ }$ is primarily due to the overlap of the resonant mode with the free-space emission spectrum of $\hbox {Alq}_3$, which peaks between 525 and 535 nm.

It is possible for the $\hbox {Alq}_{3}$ OLED to simultaneously pump two of the cavity modes. Because the peak-wavelength spacing between adjacent modes follows Eq. (1), it is difficult to have two modes simultaneously overlapping the visible-range emission spectrum for $j=1,\ 2,$ and 3. A very thick microcavity could certainly show multi-mode emission spectra. In the ARES plot for the $3\lambda /2$ microcavity shown in Fig. 4, the $3\lambda /2$ mode shows a strong normal emission peak at 485 nm. Assuming perfect mirrors, Eq. (1) leads to an estimate that the $\lambda$ mode resonant wavelength should be located at 727 nm. The emission intensity of $\hbox {Alq}_{3}$ at 727 nm and above is extremely small, and indeed we measure no normal emission upwards of 700 nm. However, as the microcavity is viewed from increasingly higher angles, the emission from the $3\lambda /2$ mode shifts from blue to violet and then vanishes entirely at angles over $55^{\circ }$ as the resonant mode no longer overlaps the $\hbox {Alq}_{3}$ emission. Starting near $30^{\circ }$ emission angle, the swallow-tail splitting of the $\lambda$ mode emission becomes evident as additional red emission. This demonstrates multi-mode pumping which is only evident here at higher viewing angles.

In semiconductor microcavities, such as the OLED devices studied here, it is possible for the coupling of the molecular exciton to the cavity photon mode to exceed the decay rate. This strong-coupling regime is characterized by resonant oscillation of energy between the exciton and photon, resulting in a Rabi splitting of the energy modes given by:

10 $$\begin{aligned} \Omega =2\sqrt{V^2+\frac{1}{4}[E_{\mathrm{Ex}}-E_{\mathrm{m}}-i(\gamma _{\mathrm{Ex}}-\gamma _{\mathrm{m}})]^2} \end{aligned}$$

where *V* is the interaction potential, $E_{\mathrm{Ex}}$ and $E_{\mathrm{m}}$ are the exciton and cavity photon energies, and $\gamma _{\mathrm{Ex}}$ and $\gamma _{\mathrm{m}}$ are the respective linewidths^7,31^. The Rabi splitting can be observed near the resonant condition ($E_{\mathrm{Ex}}\approx E_{\mathrm{m}}$), provided that the decay rate condition is met:

11 $$\begin{aligned} 2|V|>|\gamma _{\mathrm{Ex}}-\gamma _{\mathrm{m}}| \end{aligned}$$

Strong-coupling has been previously observed in the electroluminescence from an OLED, where the emitter consists of a J-aggregate dye emitter in a multilayer superlattice with a polyelectrolyte, resulting in highly-ordered, 1-dimensional excitons^32–34^. Such a system shows the strong emission and absorption with minimal Stokes shift and narrow linewidths comparable to the cavity mode. In this work, we see no evidence of strong coupling. $\hbox {Alq}_3$ is shows a Stokes shift of 840 meV, and as such our cavities are tuned far from the resonance condition^35^. Furthermore, we do not believe that strong coupling would be observed in transmission or reflection if the cavities were tuned to resonance at the 3.17 eV absorption peak of $\hbox {Alq}_3$, because the linewidth of the $\hbox {Alq}_3$ exciton peak is roughly 500 meV. The strong coupling condition (Eq. 11) would require a Rabi splitting in excess or 400 meV, more than double the giant Rabi splitting observed in other organic materials^7,34^.

**Figure 5:**
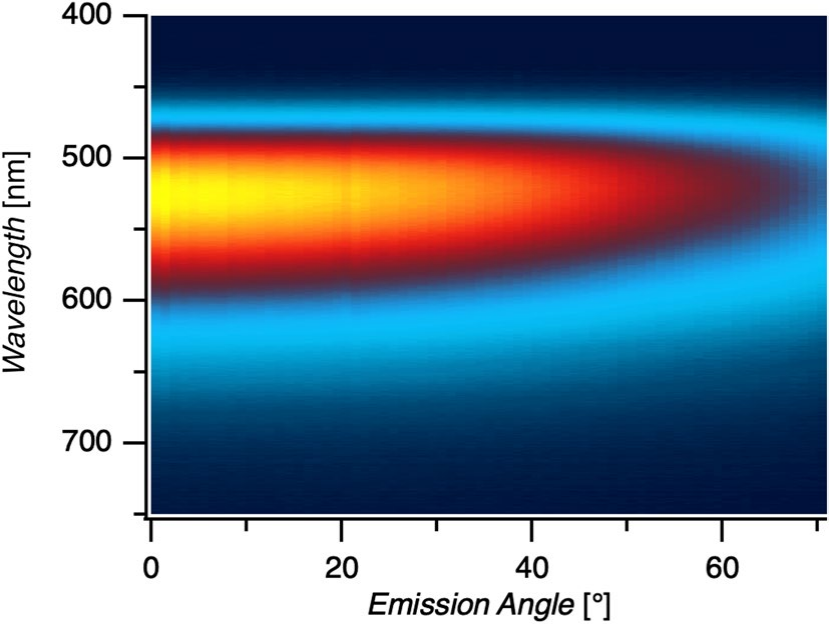
ARES data for an $\hbox {Alq}_3$ OLED of 100 nm thickness on an ITO substrate driven at $33\,\hbox { mA/cm}^{2}$.

For comparison to other OLED technologies, we note that the 113 nm thick ($\lambda /2$) device has a luminous efficacy of 0.1 lm/W at a current density of $500\,\hbox { mA/cm}^{2}$. Although luminous efficacy is a standard metric for OLED characterization, it is not well-suited to describe the narrow bandwidth, angular dependent emission observed here. Instead we note that the radiant intensity at $0^{\circ }$ emission angle when driven at $500\,\hbox { mA/cm}^2$ for the 113 nm, 282 nm, and 362 nm cavities shown in Fig. 4 are $3.4\, \upmu \hbox {W/sr}$ (10 V), $0.14\,\upmu \hbox {W/sr}$ (22 V), and $0.46\,\upmu \hbox {W/sr}$ (28 V). The peak radiant intensity of the 282 nm device is at $65^{\circ }$ viewing angle and is roughly twice as high as at $0^{\circ }$. The $\lambda$ and $3\lambda /2$ mode devices emit 10 and 8 times less light while consuming 2.2 and 2.8 times the power compared to the $\lambda /2$ mode device. The efficiency is therefore $\sim 22$ times lower because ohmic losses in the thicker devices result in excess power consumption in the form of Joule heating as will be discussed below. The strength of the dipole emitters and the outcoupling efficiency depend heavily on the position of the dipole within the microcavity, which is the subject of ongoing investigation. The ARES of the microcavity OLEDs is in stark contrast to an ITO-based OLED that shows broadband emission and no angular dispersion, as seen in Fig. 5, which shows a radiant intensity of $22.5\, \upmu \hbox {W/sr}$ at $0^{\circ }$ emission angle.

### Perceived color of emission

**Figure 6:**
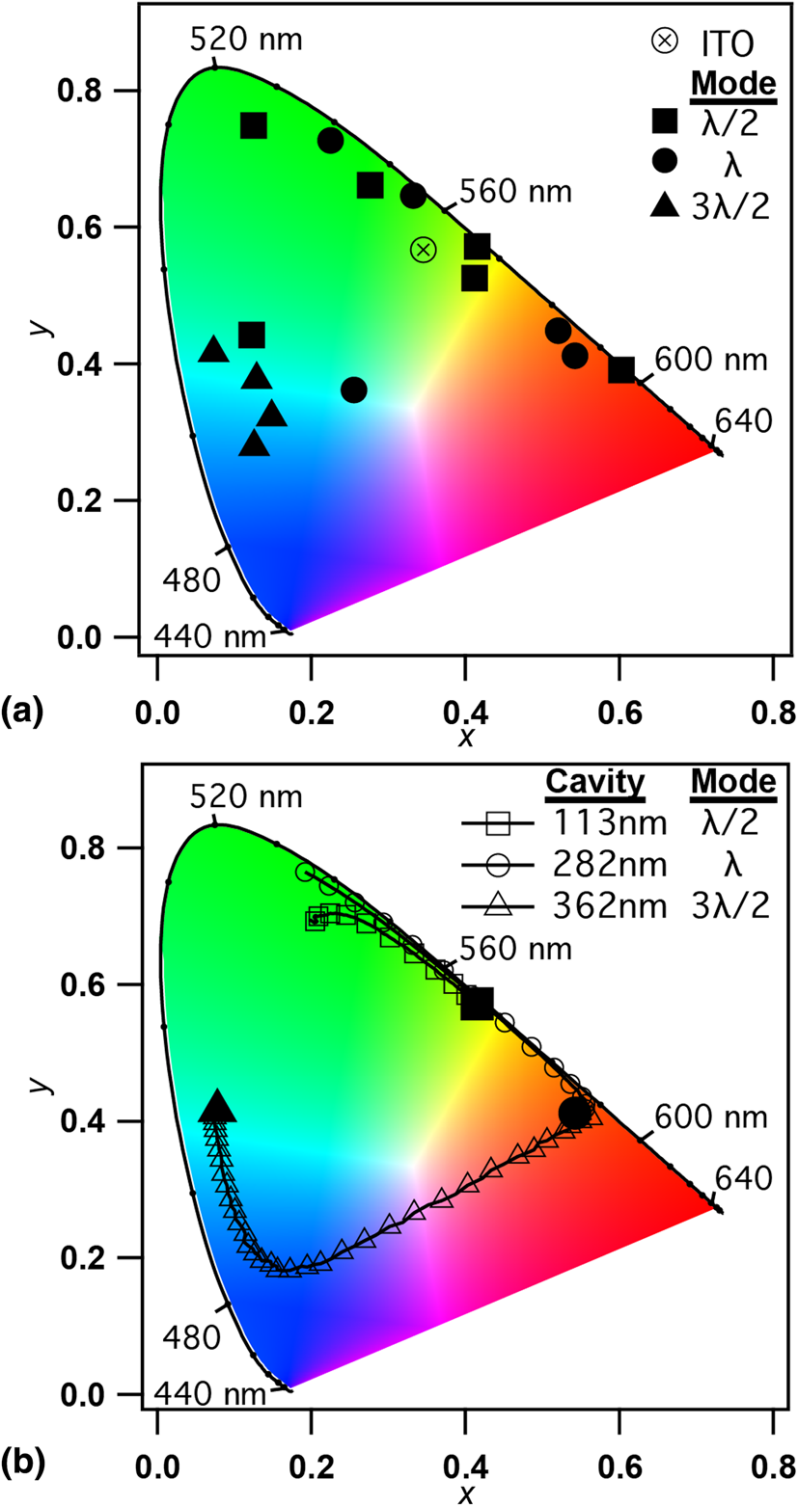
CIE 1931 color coordinates of the perceived emission from (**a**) microcavities at normal incidence with an ITO-based $\hbox {Alq}_3$ OLED indicated as the $\otimes$ symbol for reference and, (**b**) microcavity OLEDs for $\lambda /2$ mode, $\lambda$ mode, and $3\lambda /2$ mode observed at angles from $0^{\circ }$ to $70^{\circ }$. Normal emission is indicated with the solid, larger symbol and the open symbols of the same shape proceed generally counterclockwise around the color gamut with increasing angle.

The color of the microcavity OLEDs perceived by the human eye is quantified by the CIE 1931 color-matching functions. The chromaticity coordinates *x* and *y*, and the overall luminance, *Y*, are calculated from the overlap integrals of the color-matching functions with the device emission spectrum. The chromaticity coordinates for the microcavity OLEDs are shown in Fig. 6. Figure 6a shows the *x*, *y* chromaticity coordinates for standard green emission from an ITO-based $\hbox {Alq}_3$ OLED, along with the normal emission of each of the microcavity spectra shown in Fig. 2. Through the three resonant modes investigated, the microcavities can be tuned to have red, orange, yellow, green, or blue emission. The green-emitting microcavities lie much closer to the spectral boundary of the CIE chromaticity gamut due to the narrow bandwidth. Figure 6b shows the *x*, *y* chromaticity coordinates for the three representative microcavity devices shown in Fig.  4, at emission angles ranging from $0^{\circ }$ to $70^{\circ }$ in $1^{\circ }$ increments. The solid symbol represents the normal emission color coordinates, and the open symbols of the same shape represent the spectra measured at higher angles. Increasing the emission angle causes the chromaticity coordinate to proceed counterclockwise around the color gamut (a general trend with measurement noise causing some scatter). The $\lambda$ mode microcavity is red/orange when viewed directly and becomes orange, yellow, then green as the viewing angle increases. The normal emission from the $3\lambda /2$ mode microcavity appears light blue, which becomes dark blue, violet, purple, and then red as the angle increases. The purple and red color change is due to the multi-mode pumping described above. The $j=1,\ 2,$ and 3 harmonic modes of the microcavity can be used to convert the broadband green emission of $\hbox {Alq}_3$ into virtually any color on the gamut, including along the line of purples but excluding the deep red and violet corners.

### Power dissipation

Pumping the higher order modes with the visible light emission of $\hbox {Alq}_3$ required fabricating abnormally thick OLED layers, with thicknesses up to 3.5 or 4 times the optimal thickness (90–110 nm) for high efficiency devices^18,36,37^. Thick OLEDs require higher operating voltages to drive the same current densities. Furthermore, the semitransparent Al top-emitting electrode has transmittance on the order of 1%, requiring higher current density to achieve comparable emission intensity^38^. The combined effects dictate that these devices are driven at high power and much of the driving electrical power goes to Joule heating of the microcavity OLED^17^.

In the pursuit of power efficiency, modern OLED devices address this heating issue through the introduction of dopants to the ETL and HTL, as well as by taking steps to ensure optimal energy band matching through materials selection^39^. However, the introduction of dopants or materials with a narrow band gap adds significant optical losses within the cavity structure. While these losses are relatively minor for lighting applications, within a microcavity structure light interacts with the materials for an extended time. The absorptive losses thus multiply and can lead to drastic reductions to the outcoupling efficiency and increases to peak linewidth according to our simulations. Red emitters require lower voltage operation and have been previously demonstrated to pump the second cavity mode ($\lambda$ mode) at low current density (0.7–$4.5\,\hbox { mA/cm}^{2}$) with quality factor of 30^40,41^. These devices show very high external quantum efficiency due to the Purcell effect in the microcavity^39^. Undoped, wide band gap ETL and HTL layers and the green-emitting $\hbox {Alq}_3$ are well-suited to fully characterize the higher order microcavity effect throughout the visible range, but require higher power.

**Figure 7:**
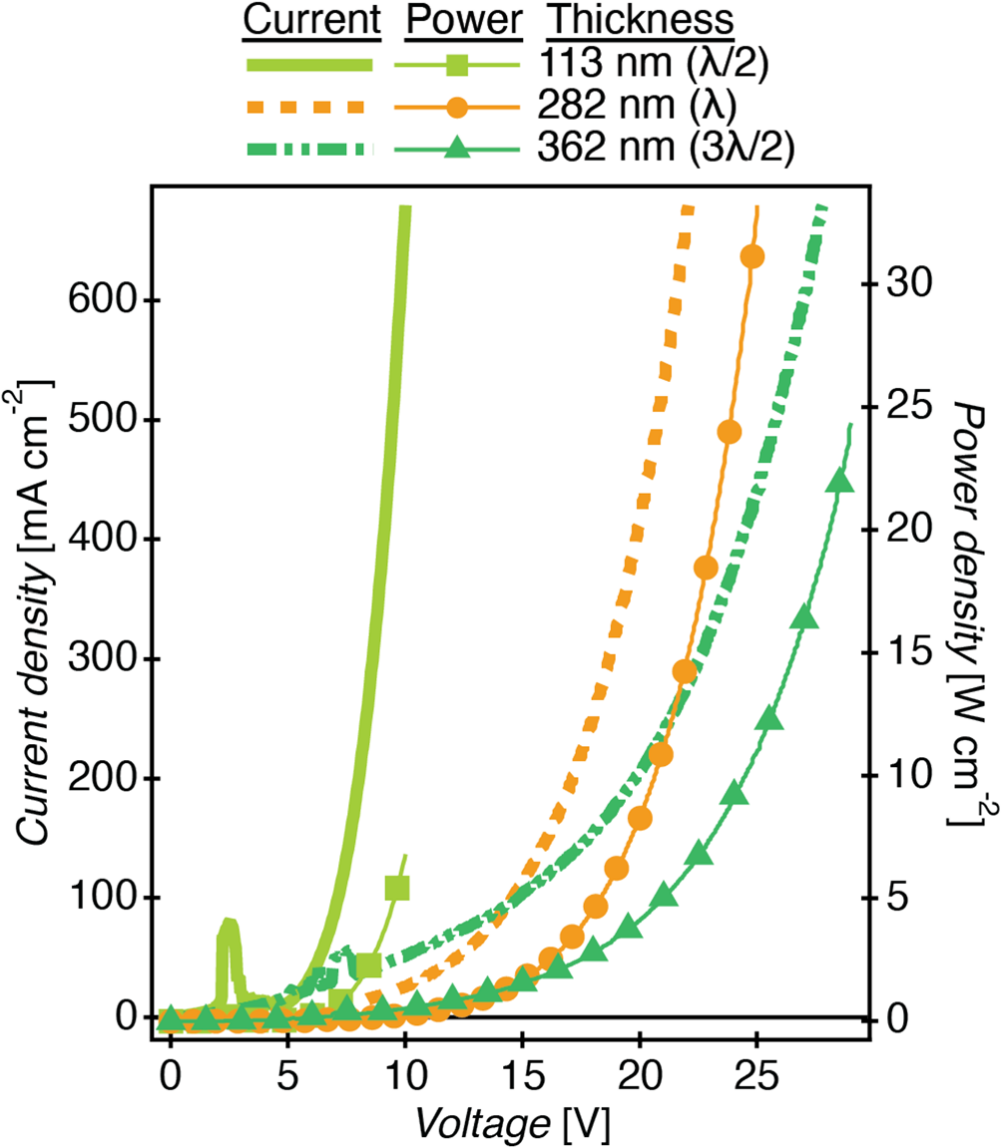
Current density (left axis, solid and dashed lines) and power density (right axis, lines with markers) vs. applied voltage for microcavity OLEDs of thickness 113 nm ($\lambda /2$), 282 nm ($\lambda$), and 362 nm ($3\lambda /2$) representing the three resonance modes. Supplementary Figure S5 shows the same data on a log-log scale.

The current density and power density of three representative microcavity OLEDs of varying thickness are shown in Fig. 7. The driving power density of the devices ranges from $5\,\hbox { to }\,30\,\hbox { W/cm}^{2}$. Bergemann et al. showed that an OLED on glass with no additional external heat sink will reach a steady state temperature of $85\,^{\circ }\hbox {C}$ when driven at $0.1\,\hbox { W/cm}^{2}$, 50–300 times less than the power consumption of these microcavity OLEDs^17^. The top-emitting devices require that the substrate itself must act as (or couple to) a heat sink, and therefore glass cannot be used as the substrate material. Our microcavity OLEDs were fabricated on silicon wafer substrates, which have thermal conductivity roughly 100 times that of glass, allowing the substrate to effectively dissipate excess heat away from the microcavity during high-power operation^20,21^. Using a thermal circuit model for one-dimensional heat transfer, we estimate that that the silicon wafer substrate reduces the equilibrium temperature of the microcavity OLEDs (driven at $4.44\,\hbox { W/cm}^{2}$) to $66\,^{\circ }\hbox {C}$ compared to $110\,^{\circ }\hbox {C}$ on glass. The equilibrium temperature is largely independent of the microcavity thickness, as the $<300$ nm thick organic layers do not constitute the largest thermal impedance. See Supplementary Figure S6 and the related discussion for further details. Silicon brings the additional benefit of planarity, as lower surface roughness leads to a more uniform microcavity thickness. Breakdown due to Joule heating is observed in the thinner ($\lambda /2$ mode) microcavity devices at current densities in excess of $1500\,\hbox { mA/cm}^{2}$ and power densities in excess of $30\,\hbox { W/cm}^{2}$. Below this threshold, the devices are functionally stable. They can be operated under DC power in excess of $10\,\hbox { W/cm}^{2}$ for over 2 min with $<2\%$ change in current density at a given driving voltage and can be stored safely in a nitrogen atmosphere for re-use. A full characterization of the shelf and operational lifetimes of the microcavities is beyond the scope of this work.

## Conclusions

This work represents an experimental and computational characterization of the optical emission from microcavity OLEDs with thickness ranging from 99 to 362 nm. Direct electrical pumping of higher-order harmonic modes is demonstrated, and the dependence of the resonant wavelength, the bandwidth (quality factor), and polarization on the cavity thickness, the viewing angle, and the mode number are measured. The resonant peak wavelength traverses the visible range for the fundamental and second harmonic mode, and the thickest microcavities show blue emission in the third harmonic mode. Accessing the higher modes requires a silicon wafer functioning both as a planar substrate and heat sink, enabling high-power operation. The color of the emission changes when viewed from different angles, shifting towards the blue with increasing angle. The microcavity effect and the simultaneous pumping of multiple cavity modes allow the $\hbox {Alq}_3$ OLEDs to emit almost any color including those along the purple line, functionally spanning the color gamut. A computational modeling tool was developed that predicts all of the observed optical phenomena.

Microcavity OLEDs represent a promising building block for photonic applications including novel monochromatic and tunable white light sources comprising coupled microcavity OLEDs, which may be useful for solid state lighting or multi-wavelength communication systems^42,43^. A thorough characterization of the resonant modes beyond the photonic ground state and a predictive computational modeling tool will enable the design of complex device structures that may rely on pumping, sampling, or mixing of these higher order harmonic modes.

## Supplementary Information

Supplementary Information 1.
