# Supplementary material for: Characterization of higher harmonic modes in Fabry–Pérot microcavity organic light emitting diodes
Source: Sci Rep. 2021 Apr 19;11:8456. doi: 10.1038/s41598-021-87697-8 (PMC8055888; doi:10.1038/s41598-021-87697-8)
Supplement: Supplementary file 1 — Supplementary Information 1. [file 41598_2021_87697_MOESM1_ESM.pdf]

Supplementary Information

Characterization of Higher Harmonic Modes in Fabry-Pérot Microcavity Organic Light Emitting Diodes

Ekraj Dahal[1], David Allemeier[1], Benjamin Isenhart[2], Karen Cianciulli[3], and Matthew S. White[2,1,*]

[1]Materials Science Program, University of Vermont, 82 University Pl., Burlington, VT 05405
[2]Department of Physics, University of Vermont, 82 University Pl., Burlington, VT 05405
[3]Asheville School, 360 Asheville School Rd., Asheville, NC 28806

[*]Corresponding author: mwhite25@uvm.edu

Quantitative Details of Standing-Waves in Resonant Modes:

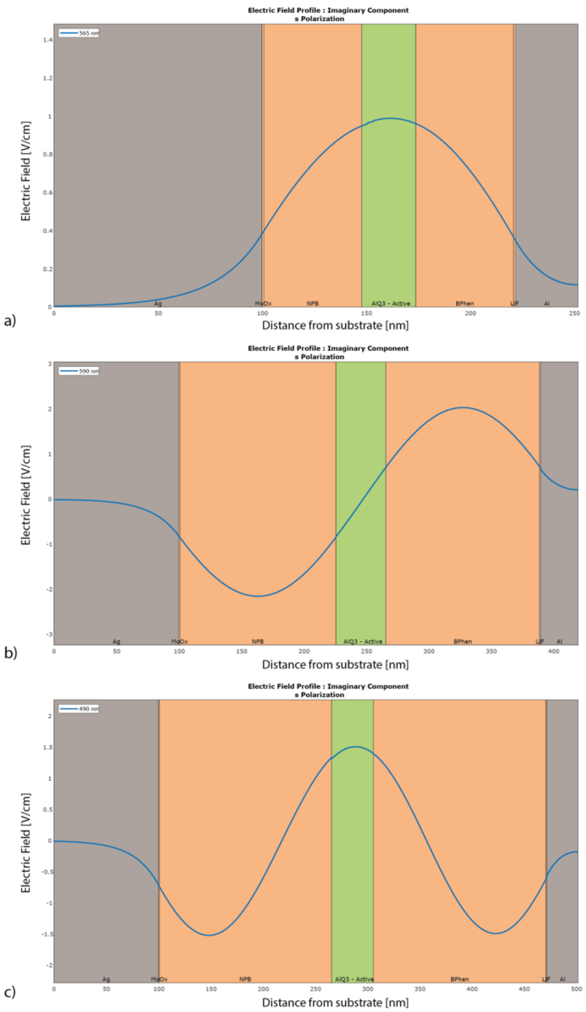

Figure S1: Electric field distribution as simulated according to the procedures described below for the a) λ/2, b) λ, and c) 3λ/2 resonant modes. These field profiles were used in the schematic Figure 1a.

Overview of OLED Transfer Matrix Simulations:

In order to optimize the OLED device structure and explore variations on its design, a numerical simulation program was developed in Matlab following the excellent work of Benisty et al., Lukosz, and Chance, Prock and Silby. [1-4] The basis of this model is the transfer matrix method, an elegant and commonly used formulation of the optical behavior of two-dimensional planar structures. This method relates the incident electric field to the transmitted and reflected fields for arbitrary multilayer structures by means of a transfer matrix and can be generalized to include off-normal incidence, absorption, polarization and waveguiding. The transfer matrix method is thus well suited to the simulation of the angular emission spectrum and outcoupling efficiency of OLED devices.

Within such devices, particularly within microcavity OLEDs, there exist reflections within the structure which give rise to interference between the reflected waves and the incident waves. At each interface, the difference in index of refraction can cause some or substantially all of a wave to be reflected. Further, these reflected waves *also* undergo reflections. This complicates the calculation of the external emission spectrum and outcoupling efficiency. The solution requires solving the forward and backward propagating wave components simultaneously within each layer in the infinite reflection limit. This is accomplished by recognizing that the two field components within a layer j may be described as a superposition of two electric fields of wave vector k_j travelling in opposite directions, with amplitude $E_j^{\uparrow}$ and $E_j^{\downarrow}$, borrowing the notation of Benisty. [1] This superposition may then be represented as a vector $\boldsymbol{E_j^{\uparrow\downarrow}}$ according to equation 1.

$$\boldsymbol{E_j^{\uparrow\downarrow}} = E_j^{\uparrow} e^{i(k_j x - \omega t)} + E_j^{\downarrow} e^{-i(k_j x + \omega t)} \equiv \begin{bmatrix} E_j^{\uparrow} \\ E_j^{\downarrow} \end{bmatrix} \qquad \text{(Equation 1)}$$

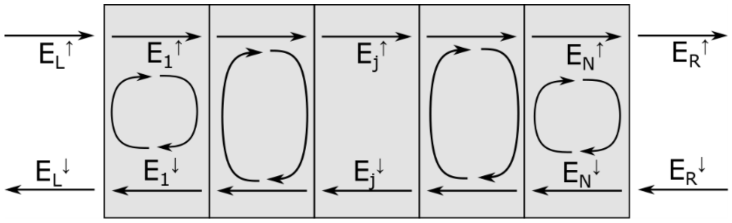

Figure S2: Schematic of the electric fields within a multi-layer stack. Within each layer is a rightward propagating wave $E_j^{\uparrow}$ and a leftward propagating wave $E_j^{\downarrow}$.

To relate the electric fields at either side of an interface, the transfer matrix method makes use of the Fresnel coefficients. These provide the reflection and transmission coefficients for light of arbitrary angle of incidence and polarization, thereby allowing exact calculation of the fractional transmittance of an electromagnetic wave at an interface. For *s*-polarized waves at normal incidence, for instance, the reflection coefficient r_{jk} between layers j and k with complex indices of refraction $\tilde{n}_j$ and $\tilde{n}_k$ is given by

$$r_{jk} = \frac{\tilde{n}_j - \tilde{n}_k}{\tilde{n}_j + \tilde{n}_k}$$

and the transmission coefficient is simply $t_{jk} = 1 - r_{jk}$. More generally, these coefficients can include the angle of incidence by Snell's law ($\tilde{n}_j \sin\theta_j = \tilde{n}_k \sin\theta_k$), and since only the far-field emission spectrum is desired, the following formulation is more convenient:

$$q_j^2 = \tilde{n}_j^2 - (\tilde{n}_{ext} \sin\theta_{ext})^2 \quad \text{and} \quad q_k^2 = \tilde{n}_k^2 - (\tilde{n}_{ext} \sin\theta_{ext})^2$$

where $\tilde{n}_{ext}$ is the index of refraction of the external medium (usually air) and θ_{ext} is the external viewing angle. This allows us to rewrite the above reflection coefficient as

$$r_{jk} = \frac{q_j - q_k}{q_j + q_k}$$

$r_{jk}^{(s)} = \frac{q_j - q_k}{q_j + q_k}$	$r_{jk}^{(p)} = \frac{\tilde{n}_k^2 q_j - \tilde{n}_j^2 q_k}{\tilde{n}_k^2 q_j + \tilde{n}_j^2 q_k}$
$t_{jk}^{(s)} = \frac{2q_j}{q_j + q_k}$	$t_{jk}^{(p)} = \frac{2\tilde{n}_j \tilde{n}_k q_j}{\tilde{n}_k^2 q_j + \tilde{n}_j^2 q_k}$

wherein the reflection coefficient now automatically considers the angle of incidence, polarization, and the absorbing properties of the two mediums. This follows the formalism of Pettersson et al., which is convenient for the purpose of modelling the far-field emission spectra for complex structures. [5]

At each interface, a system of equations relates the outgoing electric fields to the incoming fields using the Fresnel coefficients. Conservation of energy imposes the constraint $E_i = E_r + E_t$. Rearranging this system then allows us to define a scattering matrix $\boldsymbol{M_{j-1,j}}$ to relate the electric field at the interface in layer **j** to the electric field at the interface in layer **j-1**.

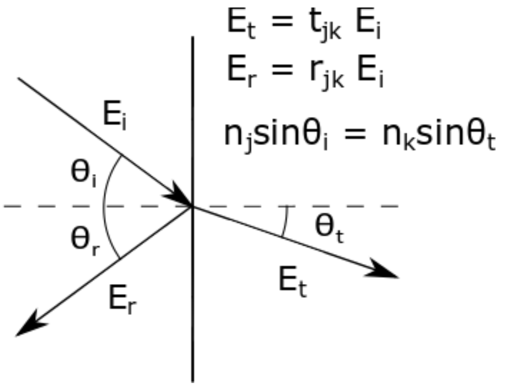

$$\boldsymbol{E_j^{\uparrow} = t_{j-1,j} E_{j-1}^{\uparrow} + r_{j,j-1} E_{j-1}^{\downarrow}}$$

$$\boldsymbol{E_{j-1}^{\downarrow} = t_{j,j-1} E_j^{\downarrow} + r_{j-1,j} E_{j-1}^{\uparrow}}$$

$$\begin{bmatrix} E_{j-1}^{\uparrow} \\ E_{j-1}^{\downarrow} \end{bmatrix} = \frac{1}{t_{j-1,j}} \begin{bmatrix} 1 & r_{j-1,j} \\ r_{j-1,j} & 1 \end{bmatrix} \begin{bmatrix} E_j^{\uparrow} \\ E_j^{\downarrow} \end{bmatrix}$$

$$\boldsymbol{M_{j-1,j}} \equiv \frac{1}{t_{j-1,j}} \begin{bmatrix} 1 & r_{j-1,j} \\ r_{j-1,j} & 1 \end{bmatrix}$$

$$\boldsymbol{E_{j-1}^{\uparrow\downarrow} = M_{j-1,j} E_j^{\uparrow\downarrow}} \qquad \textbf{(Equation 2)}$$

This formulation succinctly relates the electric fields at either side of interface **j-1/j**. In order to reach the next interface **j/j+1**, these fields must be propagated through layer **j** by means of a propagation matrix $\boldsymbol{D_j}$, which also accounts for absorption within the bulk of the layer through the complex component of the index of refraction. This requires calculation of the perpendicular wave vector component $\boldsymbol{k_{z,j}}$ within layer **j**, which obeys the wave equation [1]:

$$k_{z,j}^2 + k_{\parallel}^2 = k_j^2 = \frac{\omega^2}{c^2} n_j^2$$

The phase and amplitude shift $\boldsymbol{\varphi_j}$ for a wave propagating through a layer of thickness $\mathbf{d_j}$ may be written as:

$$\boldsymbol{\varphi_j = e^{ik_{z,j}d_j}}$$

$$\boldsymbol{D_j} = \begin{bmatrix} \boldsymbol{e^{ik_{z,j}d_j}} & 0 \\ 0 & \boldsymbol{e^{-ik_{z,j}d_j}} \end{bmatrix}$$

Now we can use the propagation matrix $\boldsymbol{D_j}$ to relate the electric field at the left and right sides of layer **j**, arriving at Equation 3.

$$\boldsymbol{E_{j^\uparrow}^{\uparrow\downarrow} = D_j E_{j^\downarrow}^{\uparrow\downarrow}} \qquad \textbf{(Equation 3)}$$

In order to traverse multiple layers, equations 2 and 3 are used repeatedly to form the 2x2 transfer matrix $\boldsymbol{S_{j\rightarrow k}}$. This allows us to express the electric field at point **k** as a function of the electric field at point **j**.

$$\boldsymbol{S_{j\rightarrow k}} = \prod_{\boldsymbol{n=j}}^{\boldsymbol{k}} \boldsymbol{D_n M_{n,n+1}} = \begin{bmatrix} \boldsymbol{S_{11}} & \boldsymbol{S_{12}} \\ \boldsymbol{S_{21}} & \boldsymbol{S_{22}} \end{bmatrix}$$

$$\boldsymbol{E_j^{\uparrow\downarrow} = S_{j\rightarrow k} E_k^{\uparrow\downarrow}} \qquad \textbf{(Equation 4)}$$

Many software packages for the analysis of multi-layer optical coatings and solar cells use the values of the **S** matrix to quantify the reflectivity and transmission through the coating. To properly calculate the emission from *within* a multi-layer stack, however, further steps must be taken. First, the source terms $\boldsymbol{A_{s,p}^{\uparrow\downarrow}}$ which quantify the angle and polarization dependent emission strength of classical dipoles must be calculated according to the equations provided by Benisty et al. [1] Next, two transfer matrices are constructed between the source plane and the right and left-hand sides of the multilayer stack. The resulting system of linear equations can be simplified to allow direct calculation of the external electric fields on the RHS and LHS using only the source terms and transfer matrix components. [1]

$$\boldsymbol{E_L^{\uparrow} = S_{L\rightarrow A} A_{s,p}^{\uparrow\downarrow}}$$

$$\boldsymbol{E_R^{\downarrow} = (S_{A\rightarrow R})^{-1} A_{s,p}^{\uparrow\downarrow}}$$

$$\begin{bmatrix} 0 \\ E_L^{\downarrow} \end{bmatrix}^{(s)} = \boldsymbol{S_L} \begin{bmatrix} A^{\uparrow} \\ A^{\downarrow} \end{bmatrix}^{(s)} \qquad \begin{bmatrix} E_R^{\uparrow} \\ 0 \end{bmatrix}^{(s)} = \boldsymbol{S_R} \begin{bmatrix} A^{\uparrow} \\ A^{\downarrow} \end{bmatrix}^{(s)}$$

$$E_L^{\downarrow} = \frac{S_{22}^L A^{\uparrow} + S_{12}^L A^{\downarrow}}{S_{11}^R S_{22}^L - S_{21}^R S_{12}^L} \quad \textbf{(Equation 5)}$$

Using this starting point, the program developed for this project provides a full simulation of the angle and polarization-resolved emission from within a multi-layer stack. Right-hand and left-hand transfer matrices are calculated for each polarization and angle and are used to calculate the external field strength relative to the normalized dipole source terms located on a plane within the source layer. These calculations are repeated for multiple source plane positions and combined to simulate spatially distributed emission within the active layer. These planes are then weighted by an exponential exciton concentration profile. [5] Further, linear interfacial scattering due to surface roughness is incorporated into the scattering matrix **M** (not shown above). Finally, the output spectrum is weighted by applying an electroluminescence spectrum of Alq_3 to account for the free-space excitonic energy distribution.

The simulations assume an exciton diffusion length of 3 nm which results in a weighted dipole emission closer to the HTL/EML interface in accordance with experimental results. [6] RMS surface roughness values were taken to be between 0 and 8 nm for the ETL, which were determined experimentally by AFM measurements of BPhen films deposited on silicon. Ten dipole layer positions were used to calculate the final outcoupling efficiency, which were evenly distributed through the EML. The broadband emission spectrum of Alq_3 with a peak at 526 nm was applied to the results to account for the wavelength-dependent emission strength. An anisotropic distribution of dipole orientations (30:1 horizontal to vertically oriented dipoles) was used to achieve the best correspondence with experimental results. This value of anisotropy was kept constant for all computational experiments.

Summary of Simulation Parameters for the Figures

Table S1: Layer thicknesses used in simulation for theory/experiment comparison.

Device	Layer Thicknesses (nm)							
	Silicon	Ag	MoOx	NPB*	Alq3	BPhen*	LiF	Al
λ/2	0	100	1	39	20	60	1	30
λ	0	100	1	102	40	147	1	30
3λ/2	0	100	1	133	40	192	1	30

* Nominal thicknesses for NPB and BPhen were 45, 120 and 150nm. Actual layer thicknesses for NPB were retroactively established by calibration tests, and the thickness of BPhen was taken as an experimental parameter to achieve best fit due to lack of calibration data.

Table S2: Summary of simulation parameters. Parameters were kept constant for all figures.

Parameter	Value
Dipole Calculation Mode	Uniform distribution across active layer
Number of Dipole Layers Calculated	10
Exciton Spatial Distribution	Exponential
Exciton Diffusion Length	3 nm
Dipole Orientation Ratio (H:V)	30:1
Emission Correction	Yes
Emission Offset	0.2

Polarization of Emission:

When viewing the emission from microcavity OLEDs from an incident angle that deviates from normal, the emission pattern has distinct polarization characteristics. The swallow-tail splitting results from the different boundary conditions at the conducting top mirror enforced by Maxwell's equations for the s and p-polarizations. For Fabry-Pérot microcavities, this effect is primarily due to the polarization-dependent phase shift at the metal surfaces. [7] This is confirmed by inserting a linear polarizer in the detector path at 0° (s-polarized) or 90° (p-polarized) with respect to the viewing angle axis of rotation. Supplementary Figure S3 shows the unpolarized, 0° (s-polarized), and 90° (p-polarized) emission from three microcavities with thicknesses that allow pumping of the first three cavity modes. The higher-energy branch of the swallow-tail splitting is clearly s-polarized, and the lower-energy branch p-polarized. It is also apparent that the p-polarized emission is relatively stronger at higher viewing angles. This is confirmation of the expected behavior based on classical dipole emission within a cavity.

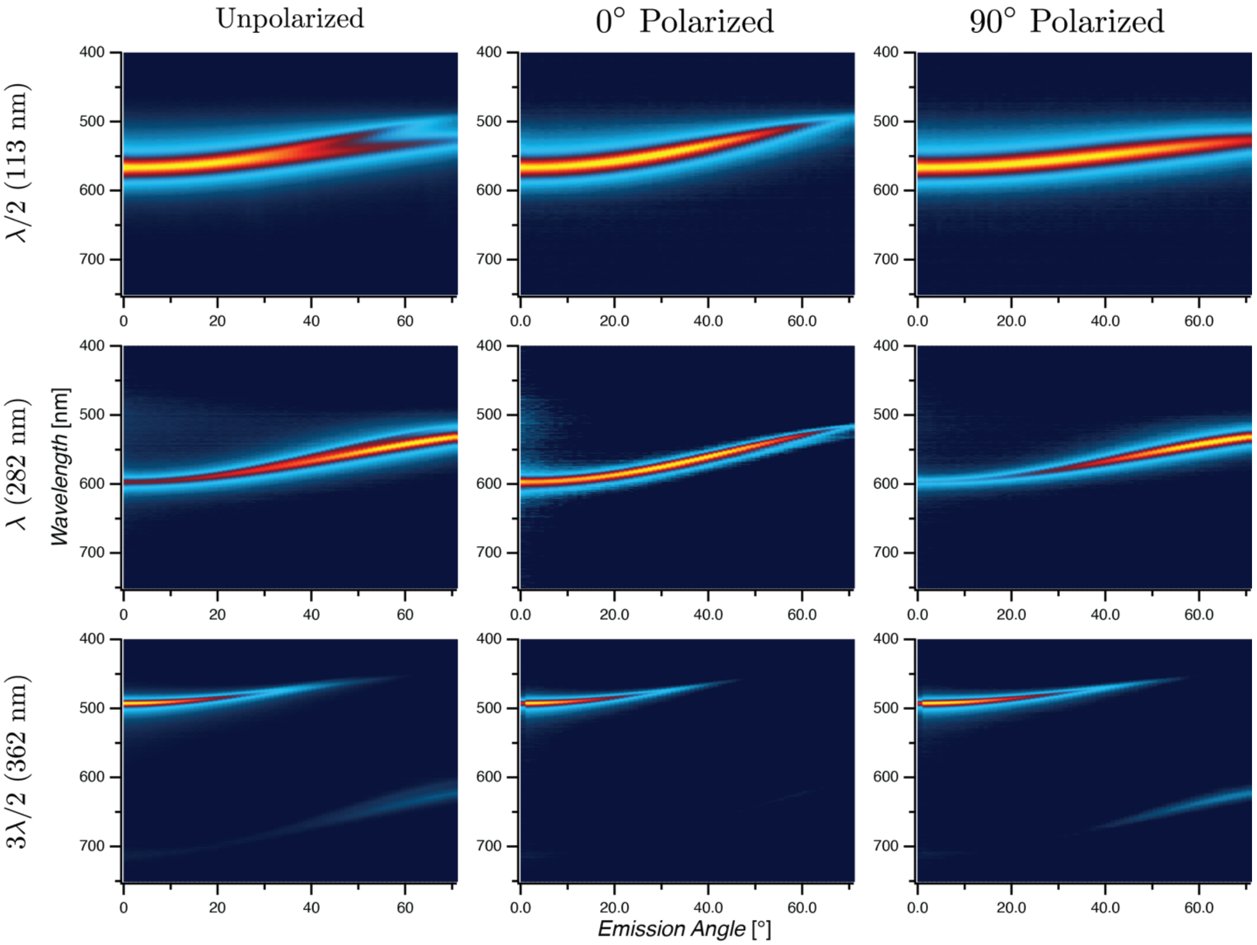

Figure S3. Experimental angle-resolved electroluminescent spectra for microcavity devices with cavity thickness 113 nm representing the λ/2 mode, 282 nm the λ mode, and 362 nm the 3λ/2 mode. The left-most spectral matrix is the same as shown in Figure 4 of the primary text, with no polarizer in the detector. The middle column shows spectral matrices with a polarizer oriented parallel to the viewing-angle axis of rotation (0° s-polarized). The right column spectra were measured with the polarizer oriented perpendicular to the viewing-angle axis of rotation (90° p-polarized).

As discussed by Benisty[1], the relative strength of the s and p polarizations at a particular angle is determined by the ratio of the vertical to horizontal components of emitting dipoles within the source plane. Horizontal dipoles, which are oriented in the plane of the microcavity structure, emit an equal proportion of s and p polarized light along the normal direction, falling off equally with the cosine of the angle. This behavior is clearly observed in the 3/2 λ device above. The vertical dipole components, however, emit purely p polarized light with a strength that depends on the sine of the angle. The high-angle emission observed in the λ device is due to the dominance of the vertical dipole components. This is evidenced by the lack of s polarization at high angles, despite seeming of equal intensity due to normalization.

Normalized emission source terms based on classical dipole emitter patterns can be found in [1] which capture these contributions.

Reflectance of Metal Electrodes:

The reflectance of the individual electrode mirrors was simulated using the transfer matrix method described above. We see that the product of the reflectance R1 × R2 depends on the wavelength, but is roughly 0.8 throughout the visible range.

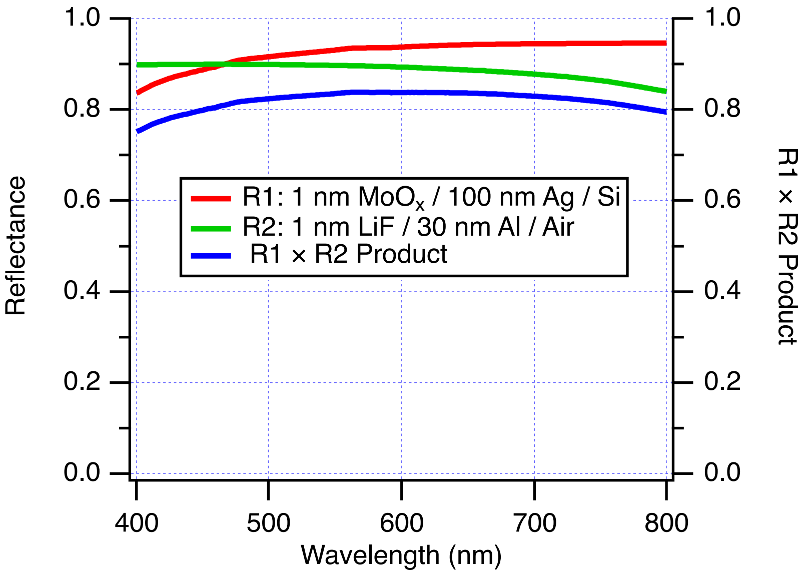

Figure S4. Simulated reflectance of the bottom mirror (R1) consisting of 1 nm MoO_x on 100 nm Ag on Si and a top mirror (R2) consisting of 1 nm LiF on 30 nm Al outcoupling to air. The blue line represents the R1 × R2 product, the relevant quantity in estimating the cavity Q factor.

Electrical Characterization:

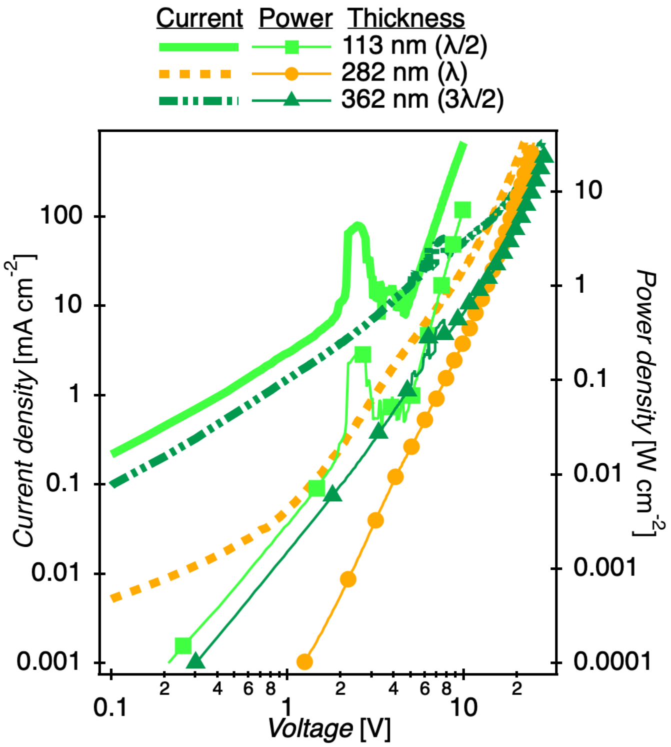

Figure S5. Log-log plot of the current density and power density vs. voltage of three representative devices.

Thermal Equilibrium of Microcavity OLEDs

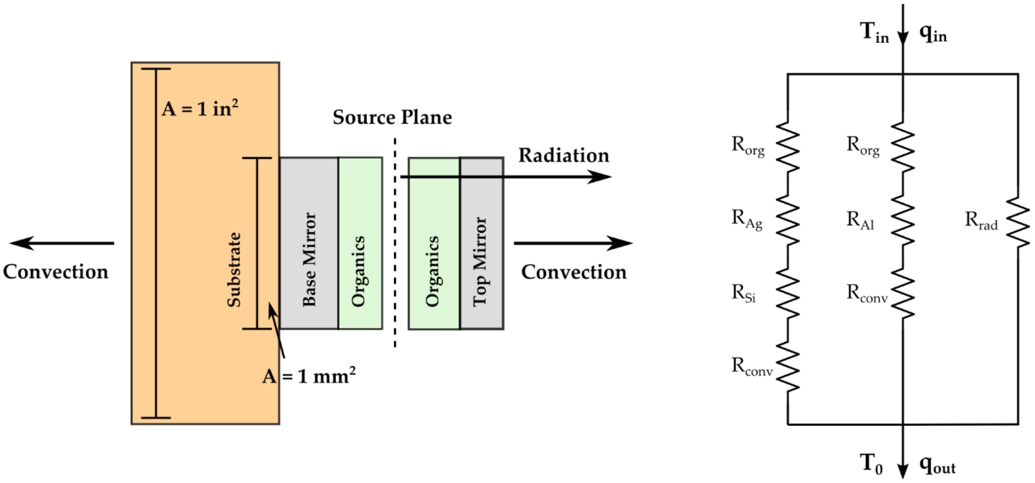

Figure S6: Basic thermal circuit model for a microcavity OLED. Heating is assumed to occur on a plane at the HTL/EML interface, where most exciton generation and recombination is occurring. Equilibrium is established by heat transfer from the OLED into a surrounding column of ambient air through conduction and convection. Thermal gradients across the surface of the substrate were ignored for simplicity.

An estimation of the operating temperature of the microcavity OLEDs was conducted using a thermal circuit model. The microcavity was assumed to be in equilibrium due to convective and radiative heat transfer ($h_{rad} \approx 5$ W/m^2 K)[8] from the top and bottom of the device into ambient air conditions, with heat input equaling the outflow. A heat conversion efficiency of over 99% was calculated through comparison of the electrical power input to the integrated radiant intensity of the OLED microcavities as measured by a calibrated photodiode.

Table S3: Summary of parameters for thermal simulation. The 120nm microcavity device was chosen as the basis of the study.

Layer	Thickness	Thermal Resitivity (W/K·m)	Thermal Resistance (K·m^2/W)
Top Air Pocket	1.5 cm	0.025 [8]	0.6
Aluminum Top Mirror	30 nm	20 [8]	1.50×10^{-9}
Top Organic Layers	47 nm	0.2 [8]	2.35×10^{-7}
Bottom Organics Layers	47+26 nm	0.2 [8]	3.65×10^{-7}
Silver Bottom Mirror	100 nm	429 [9]	2.33×10^{-10}
Silicon Substrate	500 µm	125 [10]	4.00×10^{-6}
Glass Substrate	1 mm	1 [11]	1.00×10^{-3}
Bottom Air Pocket	1.5 cm	0.025 [8]	0.6

$$q_{in} = q_{out} = \frac{\Delta T}{R_{eff}} \qquad \frac{1}{R_{eff}} = \sum_{p\,(paths)} \frac{1}{R_p} \qquad R_{path} = \sum_i \frac{t_i}{k_i} \quad R_{rad} = \frac{1}{h_{rad}} \qquad R_{conv} = \frac{1}{A_{sub}} R_p^{air}$$

Table S4: Estimated operating temperatures of OLED microcavities with 50 mW applied power.

Device	ΔT (K)	Temperature (°C)
120nm MC OLED on Si	41.2	66.2
120nm MC OLED on Glass	84.6	109.6
354nm MC OLED on Si	41.3	66.3
354nm MC OLED on Glass	84.7	109.7

References:

[1] H. Benisty, M. Mayer, R. Stanley, Method of source terms for dipole emission modification in modes of arbitrary planar structures, J. Opt. Soc. Am. a, JOSAA. 15 (1998) 1192–1201. doi:10.1364/JOSAA.15.001192.

[2] W. Lukosz, Light emission by magnetic and electric dipoles close to a plane dielectric interface. III. Radiation patterns of dipoles with arbitrary orientation, J. Opt. Soc. Am., JOSA. 69 (1979) 1495–1503. doi:10.1364/JOSA.69.001495.

[3] W. Lukosz, R.E. Kunz, Light emission by magnetic and electric dipoles close to a plane interface. I. Total radiated power, J. Opt. Soc. Am., JOSA. 67 (1977) 744. doi:10.1364/JOSA.71.000744.

[4] R.R. Chance, A. Prock, R. Silbey, Lifetime of an emitting molecule near a partially reflecting surface, J Chem Phys. 60 (1974) 2744–2748. doi:10.1063/1.1681437.

[5] L.A.A. Pettersson, L.S. Roman, O. Inganäs, Modeling photocurrent action spectra of photovoltaic devices based on organic thin films, J. Appl. Phys. 86 (1999) 487–496. doi:10.1063/1.370757.
[6] J. Shinar, R. Shinar, Organic light-emitting devices (OLEDs) and OLED-based chemical and biological sensors: an overview, J Phys D Appl Phys. 41 (2008) 133001. doi:10.1088/0022-3727/41/13/133001.
[7] G.H. Lodden, R.J. Holmes, Polarization splitting in polariton electroluminescence from an organic semiconductor microcavity with metallic reflectors, Appl. Phys. Lett. 98 (2011) 233301–4. doi:10.1063/1.3599058.
[8] X. Qi, S. Forrest, Thermal analysis of high intensity organic light-emitting diodes based on a transmission matrix approach, J. Appl. Phys. 110, 124516 (2011)
[9] www.goodfellow.com, Silver - online catalogue source.
[10] Virginia Semiconductor, Inc., Basic Mechanical and Thermal Properties of Silicon.
[11] Knittel Glass, Microscope Slides Data Sheet.
